# Endometrial response to conceptus-derived estrogen and interleukin-1β at the time of implantation in pigs

**DOI:** 10.1186/s40104-018-0259-8

**Published:** 2018-06-06

**Authors:** Hakhyun Ka, Heewon Seo, Yohan Choi, Inkyu Yoo, Jisoo Han

**Affiliations:** 1 Department of Biological Science and Technology, Yonsei University, Wonju, 26493 Republic of Korea; 2 Department of Veterinary Integrated Biosciences, Texas A&M University, College Station, TX 77843-2471 USA; 3 Department of Obstetrics and Gynecology, University of Kentucky College of Medicine, Lexington, Kentucky 40536-0298 USA

**Keywords:** Conceptus, Endometrium, Estrogen, Interleukin-1β, Pig, Uterus

## Abstract

The establishment of pregnancy is a complex process that requires a well-coordinated interaction between the implanting conceptus and the maternal uterus. In pigs, the conceptus undergoes dramatic morphological and functional changes at the time of implantation and introduces various factors, including estrogens and cytokines, interleukin-1β2 (IL1B2), interferon-γ (IFNG), and IFN-δ (IFND), into the uterine lumen. In response to ovarian steroid hormones and conceptus-derived factors, the uterine endometrium becomes receptive to the implanting conceptus by changing its expression of cell adhesion molecules, secretory activity, and immune response. Conceptus-derived estrogens act as a signal for maternal recognition of pregnancy by changing the direction of prostaglandin (PG) F_2α_ from the uterine vasculature to the uterine lumen. Estrogens also induce the expression of many endometrial genes, including genes related to growth factors, the synthesis and transport of PGs, and immunity. IL1B2, a pro-inflammatory cytokine, is produced by the elongating conceptus. The direct effect of IL1B2 on endometrial function is not fully understood. IL1B activates the expression of endometrial genes, including the genes involved in IL1B signaling and PG synthesis and transport. In addition, estrogen or IL1B stimulates endometrial expression of IFN signaling molecules, suggesting that estrogen and IL1B act cooperatively in priming the endometrial function of conceptus-produced IFNG and IFND that, in turn, modulate endometrial immune response during early pregnancy. This review addresses information about maternal-conceptus interactions with respect to endometrial gene expression in response to conceptus-derived factors, focusing on the roles of estrogen and IL1B during early pregnancy in pigs.

## Background

A high rate of embryonic mortality occurs in all mammals. In pigs, embryonic mortality before day (d) 30 of pregnancy can be up to 40%, and most embryonic losses occur during the peri-implantation period [1]. An understanding of the cellular and molecular mechanisms underlying conceptus–endometrial interactions for the establishment of pregnancy is essential to reducing embryonic mortality. In pigs, the establishment of pregnancy is a complex process that requires well-coordinated interactions between the implanting conceptus (embryo/fetus and associated extraembryonic membranes) and the maternal uterus. This leads to an extended lifespan for the corpus luteum (CL) for continued production of progesterone in the ovary and the secretion of histotrophs and immune modulation for conceptus development and placentation in the endometrium [2, 3].

During the peri-implantation period, the porcine conceptus undergoes dramatic morphological changes from spherical (3 to 10 mm in diameter) to ovoidal to tubular (10 to 50 mm in length) and then to filamentous forms (100 to 800 mm in length) as it secretes a variety of factors, including estrogens and cytokines, interleukin-1β2 (IL1B2), interferon-γ (IFNG), and IFN-δ (IFND), into the uterine lumen. It also migrates in the uterine lumen for appropriate embryo spacing and uses noninvasive implantation to develop a true epitheliochorial placenta [2, 4, 5]. Meanwhile, the endometrium, which is affected by progesterone from the ovary during this period, prepares for conceptus implantation by producing histotrophs such as growth factors, ions, amino acids, monosaccharides, enzymes, nutrient binding proteins, and extracellular matrix (ECM) proteins and by changing the gene expression, cellular morphology, and maternal immune environment to allow the adhesion of the conceptus trophectoderm to the endometrial epithelial cells and the development of an allogeneic fetus [3, 6, 7].

Conceptus-derived factors affect various aspects of endometrial function. Estrogens and IL1B2 are produced by the elongating conceptus on d 10–12 of pregnancy [2, 3]. Estrogens signal a maternal recognition of pregnancy in pigs because they act on a redirection of endometrial prostaglandin (PG) F_2α_ secretion from the uterine vasculature to the uterine lumen to protect the corpus luteum and ensure continued production of progesterone [2, 8]. Estrogens also affect the expression of endometrial genes involved in PG production, calcium movement, and IFN signaling [2, 9–11]. The direct effect of conceptus-derived IL1B2 at the maternal–conceptus interface is not fully understood, but it has been shown that IL1B induces the expression of many endometrial genes related to PG production and transport and the IL1B and IFN signaling pathways [10, 12, 13]. On d 12–20 of pregnancy, the conceptus trophectoderm produces significant amounts of IFN-γ (IFNG) and IFN-δ (IFND) with the highest antiviral activity on d 14–d 16 of pregnancy in pigs [14–16]. IFNG is the predominant type II IFN, comprising approximately 75% of antiviral activity in uterine flushings, and IFND is a novel type I IFN in pigs [14–16]. Unlike IFN-τ (IFNT), a type I IFN produced by the conceptus and acting as a signal for maternal recognition of pregnancy by preventing endometrial production of luteolytic PGF_2α_ in ruminants [17], IFND and IFNG do not have an anti-luteolytic effect in pigs [18]. IFNs secreted by the conceptus trophectoderm induce many IFN-stimulated genes and class I and II major histocompatibility complex (MHC) molecules in the endometrium [19–22], but detailed function of IFNs at the maternal-conceptus interface is not fully understood in pigs.

Several recent reviews have well described the events and the molecules involved in the establishment of pregnancy during the peri-implantation period in pigs [2, 9, 23, 24]. The present review highlights current information, focusing on the roles of conceptus-derived estrogen and IL1B during the implantation period in pigs.

## Estrogen, progesterone, and their teceptors during the estrous cycle and early pregnancy

**Fig. 1:**
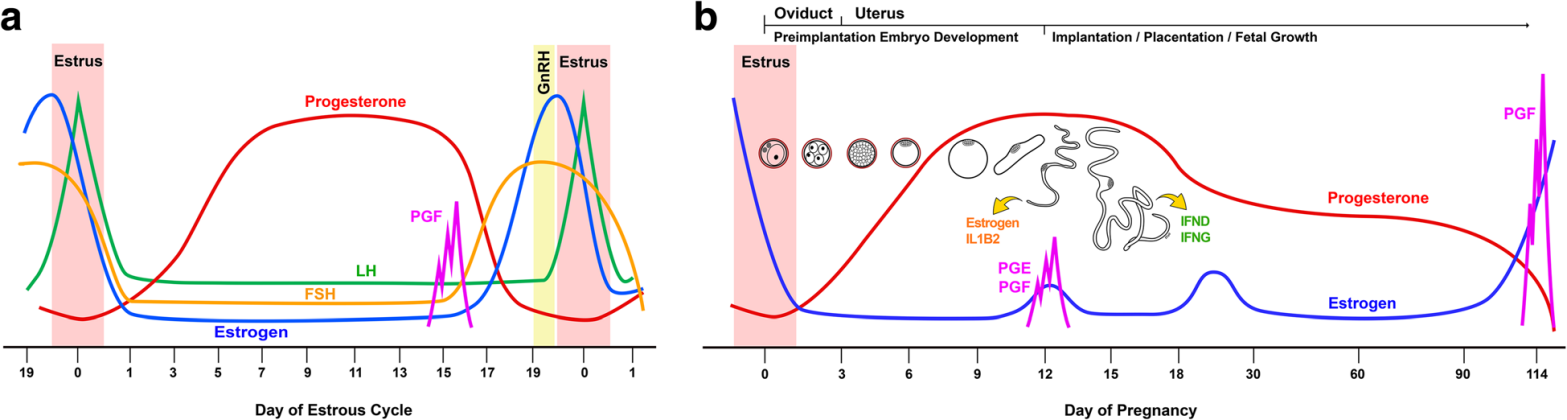
Profiles of major hormones in the blood during the estrous cycle (**a**) and pregnancy (**b**) in pigs. **a**. During the estrous cycle estrogen concentrations increase prior to estrus by the coordinated actions of gonadotropin-releasing hormone (GnRH), follicle stimulating hormone (FSH), and luteinizing hormone (LH) and decrease on the day of estrus. Progesterone concentrations increase rapidly on the day of estrus until d 12–d 14 and decrease rapidly from d 15 of the estrous cycle due to regression of the corpus luteum induced by prostaglandin (PG) F_2α_ (PGF) from the endometrium. **b.** During pregnancy estrogen concentrations decrease from estrus, maintain low concentrations with brief increases on around d 12 and d 25–d 30 of pregnancy, and increase prior to parturition. Progesterone concentrations increase from estrus to reach maximum concentrations on d 12–d 14, then decrease slowly until d 30, and remain fairly constant throughout pregnancy until near term. Developmental processes that occur in the female reproductive tract and morphological changes of preimplantation embryos and early stage conceptuses to corresponding days of pregnancy are indicated on top. Elongating conceptuses on around d 12 of pregnancy secrete estrogen and interleukin-1β2 (IL1B2), and the implanting conceptuses produce maximum levels of interferon-δ (IFND) and IFN-γ (IFNG) on around d 14–d 16. The endometrium and conceptus produce PGs on d 12, and the endometrium produces PGF to induce parturition at term

The estrous cycle and establishment and maintenance of pregnancy are regulated by the orchestrated actions of various hormones from hypothalamus, pituitary, ovary, uterus and conceptus. These hormones include gonadotropin-releasing hormone (GnRH) from the hypothalamus, follicle stimulating hormone (FSH) and luteinizing hormone (LH) from the pituitary, estrogen and progesterone from the ovary, estrogen from the conceptus and PGF_2α_ from the uterus (Fig. 1).

### Estrogen

Plasma estrogen concentrations in pigs increase prior to estrus and decrease on the day of estrus. During the estrous cycle, the mean plasma concentrations of estradiol are less than 20 pg/mL until d 16 or d 17, and then they increase to their maximal concentration of 50 pg/mL 1 or 2 d prior to estrus [25, 26]. Between d 12 and d 15 of the estrous cycle, estrone and estradiol concentrations are elevated in cyclic pigs [27]. There is no difference in plasma estradiol concentrations between cyclic and pregnant pigs for the first two weeks after the onset of estrus [25], but the estradiol concentrations in the utero-ovarian vein between d 12 and d 17 are higher in pregnant pigs than in cyclic pigs [28] (Fig. 1). Estrogen concentrations in the uterine lumen are estimated by analyzing uterine flushing from pigs [27, 29, 30]. In cyclic pigs, estrone and estradiol contents are constant at 200 to 300 pg between d 6 and d 16 of the estrous cycle, and estrone content increases to 1,000 pg on d 18. In pregnant pigs, estradiol content is about 300 pg until d 10 after the onset of estrus, at which point it increases to about 1,400 pg between d 10 and d 12, decreases to d 15, and then increases again on d 18. The estrone content in pregnant pigs also increases to 1,500 pg on d 8, decreases to d 12, then increases slowly to 3,700 pg on d 18 [27]. Total recoverable estrone, estradiol, and estriol in cyclic pigs do not change, whereas in pregnant pigs, total estrone and estradiol increase about 6-fold from d 10 to d 12. Total recoverable estrone sulfate and estradiol sulfate also increase from d 10 to d 12 in pregnant pigs [31]. The increase in estrogen concentrations in the uterine lumen of pregnant pigs reflects estrogen production by the conceptus, which converts androgens to estrogens [32, 33]. Catechol estrogens (2- and 4-hydroxyestradiol) are also converted from estradiol by porcine conceptuses during early pregnancy [34, 35].

### Progesterone

Progesterone is secreted by the CL, adrenal cortex, and placenta and is necessary for implantation, the regulation of uterine development, uterine secretion, mammary gland development, and lactogenesis. Plasma progesterone concentrations increase rapidly from less than 1 ng/mL on the day of estrus to about 30 ng/mL on d 12 and d 14 in both cyclic and pregnant pigs. In cyclic pigs, progesterone concentrations decrease rapidly from d 15 to less than 1 ng/mL on d 18 of the estrous cycle [25, 26]. This decrease in progesterone concentrations in cyclic pigs results from CL regression induced by PGF_2α_ from the uterine endometrium. In pregnant pigs, progesterone concentrations decrease slowly from d 14 to d 30, reaching 10–20 ng/mL, and then remain fairly constant throughout pregnancy until near term [25, 36] (Fig. 1). Progesterone is also present in the lumen of the uterus [27, 30]. Progesterone in uterine flushing increases from d 14 to d 16 and then decreases to d 18 of pregnancy [27]. Concentrations of pregnenolone, progesterone, and pregnenolone sulfate in uterine flushing between d 9 and d 15 are higher in pregnant pigs than in cyclic pigs [30].

### Receptors for estrogen and progesterone

Estrogen and progesterone actions in the uterus are primarily mediated through estrogen receptor-α (ESR1) and progesterone receptor (PGR), respectively. In pigs, the expression of ESR1 and PGR changes depending on the estrous cycle and pregnancy. Nuclear *ESR1* concentrations increase from estrus (d 0) to d 12 of the estrous cycle and then decrease by d 15. Endometrial *ESR1* mRNA expression is highest on d 10, declines by d 15, and then increases by d 18 in cyclic and pregnant pigs. However, in pregnant pigs *ESR1* remains suppressed after d 18 of pregnancy [37]. In cyclic and pregnant pigs, ESR1 proteins are localized in luminal epithelial (LE) and glandular epithelial (GE) cells and the stroma at estrus. ESR1 is detectable in LE and GE cells between d 5 and d 15 of the estrous cycle and pregnancy, whereas ESR1 in the stroma decreases markedly during this period. Between d 10 and d 12, strong ESR1 staining is detectable in LE and GE cells. On d 15, ESR1 staining decreases in LE and GE cells and then increases in LE and GE cells and the stroma on d 18 of the estrous cycle in cyclic pigs, but remains low after d 18 in pregnant pigs [37]. Estrogen receptor-β (ESR2), a subtype of nuclear estrogen receptors, is expressed in LE and GE cells in the endometrium during the estrous cycle and pregnancy and in conceptus trophectoderm on d 12 [38], but regulation and function of ESR2 is not fully understood in pigs [39, 40]. The presence of the membrane-associated estrogen receptors including membrane-bound ESR1 and G-protein coupled estrogen receptor 1 (GPER1), which activate non-genomic actions of estrogen, has been described in various tissue and cell types in several species [41, 42]. However, the expression of membrane-bound ESR1 or GPER1 has not been determined in the porcine endometrium.

PGR expression in the porcine uterus during the estrous cycle and pregnancy has been determined [43–45]. The endometrial *PGR* concentrations are highest between d 0 and d 5 of the estrous cycle, decrease by d 10 and d 11, and then remain low until the next proestrus phase. This pattern is the same in pregnant pigs until d 11 to d 12, and low abundance of endometrial *PGR* expression are maintained until d 85 of pregnancy. PGR protein is localized in LE and GE cells and the stroma between d 0 and d 5 with strong intensity. PGR in LE and GE cells declines from d 7, is not detectable in LE or superficial GE cells on d 12, and then increases by d 18 in cyclic pigs. In pregnant pigs, the pattern of PGR localization is the same as for cyclic pigs until d 12, but PGR staining in epithelial cells does not increase until the late stage of pregnancy. Stromal PGR is detectable throughout the estrous cycle and pregnancy, even though staining intensity is lower between d 5 and d 15 of the estrous cycle and pregnancy than at estrus. Stromal PGR increases on d 18 in cyclic pigs but not in pregnant pigs. PGR is localized to the myometrium throughout all day of the estrous cycle and pregnancy.

The down-regulation of PGR in uterine LE cells during the implantation period is a phenomenon common to several mammalian species, including pigs, ruminants, humans, and mice, indicating that loss of PGR in the uterine epithelial cells is a prerequisite for uterine receptivity to implantation, gene expression by uterine epithelial cells, and transport of molecules in the uterine lumen for a developing conceptus [46]. Because progesterone profoundly affects uterine receptivity for implantation, this paradox could be explained by stromal cell-derived growth factors known as progestamedins that are produced and released from uterine stromal and myometrial cells and express PGR through the action of progesterone [47, 48]. However, the presence of several membrane progesterone receptors, progesterone membrane component 1 (PGRMC1) and PGRMC2, and progestin and adipoQ receptor (PAQR) 5 to PAQR9, which are all G-protein-coupled receptors, has been shown in reproductive tissues and other tissues in humans, mice, and bovines [49–51]. Our study also shows that endometrial epithelial cells express PGRMC1, PGRMC2 and PAQRs during the estrous cycle and pregnancy in pigs (Kim and Ka, unpublished data), suggesting that those membrane progesterone receptors in endometrial epithelial cells could be responsible for progesterone actions during the progesterone-dominant period of the estrous cycle and pregnancy.

## Conceptus development during early pregnancy

In pigs, following fertilization, cleavage of the embryo occurs in the oviduct. Four-cell embryos enter the uterus approximately 48 h after ovulation, develop to the blastocyst stage by d 5, and then shed the zona pellucida on d 6 or d 7 [52–54]. Blastocysts measure less than 3 mm in diameter until d 10 with considerable variation [55]. During this period, the blastocysts secrete estrogen [32] and migrate in the uterus for spacing prior to implantation [53, 55, 56]. Shortly before implantation, between d 11 and d 12, porcine blastocysts undergo dramatic morphological changes, as described above. In contrast, morphological elongation of blastocysts does not occur in rodents or primates, and extraembryonic membranes are formed after implantation [57–59]. During the peri-implantation period, porcine conceptuses secrete a variety of molecules, such as estrogen, cytokines, PGs, growth factors, and proteases [2, 3].

The initial elongation from spherical blastocysts to filamentous conceptuses is achieved by cellular remodeling, not by cellular hyperplasia because the mitotic index and DNA contents of the conceptuses do not change during elongation [31]. The conceptus elongation process includes changes in microfilament orientation by rearrangement of the actin cytoskeleton [60, 61] and junctional complexes of trophectoderm cells and the migration of the endoderm at the tip of the epiblast [29]. It has been proposed that epiblast-derived fibroblast growth factor 4 (FGF4) is involved in communication with the trophectoderm cells by binding to FGF receptor 2 (FGFR2) and activating the mitogen-activated protein kinase (MAPK) signaling pathway in the trophectoderm cells of the spherical and ovoidal conceptuses prior to the elongation process [62]. FGF4 treatment of porcine trophectoderm cells in vitro induces cell migration and activates the protein kinase B (also known as the AKT) signaling pathway [63]. In addition, bone morphogenetic protein 4 from extraembryonic mesoderm is also involved in the cellular reorganization of trophectoderm cells during conceptus elongation [62]. Further growth and development of the conceptus during the peri-implantation period is stimulated by many growth factors and cytokines produced by the endometrium, including epidermal growth factor (EGF) [64, 65], FGF7 [66], insulin-like growth factor-1 (IGF1) [67], interleukin 6 (IL6), leukemia inhibitory factor [68], and transforming growth factor beta (TGFB) [69].

## Conceptus adhesion to the endometrium

The adhesion cascade for the implantation of a porcine conceptus to the maternal endometrium proceeds through a sequence of events: 1) hatching of the blastocyst from the zona pellucida, 2) precontact and orientation of the conceptus to the uterine LE cells, 3) apposition of the trophectoderm to the uterine LE cells, and 4) adhesion of the trophectoderm to the uterine LE cells [2, 59]. Although the initial early stages of implantation are common to all species, the invasion of the trophectoderm across the uterine LE cells and stroma does not occur in pigs, which uses non-invasive implantation and a true epitheliochorial type of placenta [70]. In pigs, attachment of the conceptus to the uterine epithelium initiates around d 13 to d 14, and full attachment is completed after d 18 [71]. Conceptus trophectoderm cells during this period are apposed closely to the uterine epithelium, and the embryonic disc region is rigidly attached to the uterine epithelium, with more distal regions of the chorion separated from the luminal surface [72].

The endometrial LE cells undergo morphological and functional changes during the adhesion phase. The apical-basal polarity of the LE cells decreases as the columnar epithelium with microvilli changes into cuboidal epithelium with a loss of microvilli [72]. Tight junctions between endometrial LE cells are in the basolateral region. In addition, the nuclei of LE cells become larger and more vesicular, and the cytoplasm is less dense and accumulated, with glycogen droplets at the basal side [71–73]. The apical surfaces of the LE cells are covered with a thick filamentous glycocalyx during the attachment phase [71, 73]. Mucin 1 (MUC1), a transmembrane mucin glycoprotein in glycocalyx, is down-regulated during the implantation period in pigs and ruminants [74, 75]. MUC1 is known to act as an anti-adhesive component between LE cells and trophectoderm cells by sterically inhibiting cell-cell and cell-ECM binding [58, 76]. Thus, it is suggested that down-regulation of MUC1 results in exposure of low-affinity carbohydrate ligand binding molecules such as selectins and galectins as well as a variety of cell adhesion molecules, including cadherins and integrins [23]. In humans and rabbits, the pattern of MUC1 expression in endometrial epithelial cells is somewhat different: MUC1 expression in LE cells increases during the receptive phase but is locally reduced at the attachment sites by cell surface proteases (sheddases) derived from the blastocyst or blastocyst-induced paracrine factors [58, 77]. It is believed that progesterone induces epithelial MUC1 expression, and down-regulation of PGR causes the disappearance of MUC1 on the uterine LE and superficial GE cells for the establishment of uterine receptivity to implantation [58].

Among many cell adhesion molecules, the roles of integrin and several ECM proteins have been well studied in the adhesion process between endometrial LE and trophectoderm cells in domestic animal species, including pigs and sheep [23, 76]. Integrins are heterodimeric glycoprotein receptors composed of non-covalently linked α and β subunits that bind to the Arg-Gly-Asp (RGD) and non-RGD amino acid sequences of various ECM components and cell adhesion molecules [76]. The activation of integrin receptors in LE and trophectoderm cells in the implantation adhesion process causes cytoskeletal reorganization and changes in gene expression for adhesion, migration, and invasion [76]. In pigs, uterine LE cells express integrin subunits α1, α3, α4, α5, αv, β1, β3, and β5; trophectoderm cells express α1, α4, α5, αv, β1, and β3; and αvβ1, αvβ3, αvβ5, α4β1, and α5β1 are localized at the attachment sites between uterine LE and trophectoderm cells [78]. Secreted phosphoprotein 1 (SPP1; also known as osteopontin), fibronectin, and vitronectin, which are ECM protein ligands for integrin receptors, are expressed in the endometrium at the time of LE and trophectoderm cell adhesion [78–80]. SPP1 is known to bind to αvβ1, αvβ3, αvβ5, and α4β1; fibronectin interacts with α4β1; and vitronectin binds mainly to αvβ3 [23, 78]. The expression of SPP1 in the endometrium is particularly induced by estrogen of conceptus origin at the uterine LE cells juxtaposed to the conceptus trophectoderm, beginning around d 12 and extending to all LE cells by d 20. High abundance of SPP1 expression is maintained at the maternal-conceptus interface throughout pregnancy [79, 81, 82]. In vitro analysis using porcine trophectoderm (pTr) cells and uterine endometrial epithelial (pUE) cells has shown that SPP1 binds directly to the αvβ6 integrin subunits of pTr cells and the αvβ3 on pUE cells, suggesting that SPP1 acts as a bidirectional bridging ligand during conceptus implantation [80]. The expression and function of SPP1 in the adhesion cascade at the uterine-conceptus interface has been shown in several species, including humans, mice, rabbits, and sheep, suggesting that the SPP1-mediated cell adhesion process for conceptus implantation is conserved across species [23]. Furthermore, latency-associated peptide (LAP), part of the TGFB complex, binds to integrin receptors αvβ1, αvβ3, and αvβ5 at the apical surfaces of uterine LE and trophectoderm cell attachments, suggesting that LAP-integrin complexes also promote conceptus attachment [83]. Overall, these findings indicate that in pigs the cell adhesion cascade between endometrial LE and conceptus trophectoderm cells during the implantation period is a complex process that involves a variety of adhesive factors.

## Maternal recognition of pregnancy

Progesterone is required for pregnancy maintenance beyond the estrous cycle in most mammals, including pigs, ruminants, rodents, and primates [84]. To sustain progesterone production from the CL and maintain a pregnancy, species use a variety of strategies to abrogate luteolysis. In general, the conceptus produces antiluteolytic signals that prevent the secretion or action of PGF_2α_ (pigs and ruminants) or that are directly luteotrophic to keep the CL secreting progesterone (primates).

Maternal recognition of pregnancy is usually defined as the rescue of the CL from undergoing luteolysis, although maternal function is altered as early as the period when the embryo is in the oviduct, and the mechanism to establish pregnancy and maintain CL function varies among species. The presence of a maternal recognition signal from pig conceptuses was predicted by studies on the effect of flushing conceptuses from uterine horns on various days of pregnancy. Removal of conceptuses from the uterus between d 4 and d 10 does not affect the CL lifespan [85], whereas flushing conceptuses from the uterus on or after d 12 increases the inter-estrous interval by 3 or more days [86]. Therefore, signals for maternal recognition of pregnancy in pigs are produced by conceptuses on about d 12 for the maintenance of pregnancy. Perry and coworkers first demonstrated that estrogen was produced by conceptuses during the period of maternal recognition of pregnancy in pigs [32]. There is considerable evidence for the antiluteolytic effects of estrogen [2]. Administration of exogenous estrogen in cyclic pigs between d 11 and d 15 extends the inter-estrous interval and decreases the concentration, peak height, and pulse frequency of PGF_2α_ release from the uterus [87]. Estrogen treatment on d 9.5, d 11, d 12.5, d 14, d 15.5 or d 14-16 of the estrous cycle results in an inter-estrous interval of about 30 d [88]. Daily treatment between d 11 and d 15 or two period treatments on d 11 and d 14 to d 16, corresponding to the pattern of estrogen production by conceptuses, prolongs CL function beyond d 60. The uterine content of total recoverable estrogens (estrone, estradiol, and estriol) in pregnant pigs increases on d 11 to d 12, declines on d 13 to d 14, and then increases after d 14 of pregnancy, whereas in cyclic pigs, estrogen concentration does not increase before d 15 of the estrous cycle when preovulatory follicles are present [30, 31].

The current theory of maternal recognition of pregnancy in the pig is the endocrine-exocrine theory [8]. It suggests that uterine endometrial cells differentially secrete PGF_2α_ or luteolysin, depending on estrogen secreted by conceptuses. In cyclic pigs, endometrial PGF_2α_ is secreted into the uterine vasculature, which is transported to the ovary to cause luteolysis on d 15 to d 16 of the estrous cycle (endocrine). However, in pregnant pigs, the uterine endometrium’s response to estrogen produced by conceptuses from d 11 and d 12 to d 15 is to secrete PGF_2α_ into the uterine lumen, where it is sequestered to exert its biological actions in the uterus or be metabolized to prevent luteolysis (exocrine) [2, 8]. Indeed, PGF_2α_ concentration in the utero-ovarian vein is significantly higher in cyclic pigs on d 13 to d 17 than in pregnant pigs [28]. This theory is also supported by a report that in cyclic pigs, total recoverable PGF_2α_ per uterine horn was 1.98 ng on d 11, 210.2 ng on d 17, and 66.2 ng on d 19 of the estrous cycle, whereas in pigs treated with estrogen between d 11 and d 15, total recoverable PGF_2α_ was 1.9 ng, 4,144.3 ng, and 4,646.7 ng on the same respective days [87]. PGE_2_ concentrations in the uterine lumen also increase on d 11 to d 14 in pigs [31]. In contrast to PGF_2α_, PGE_2_ could have a luteotrophic effect and protect the CL against the luteolytic action of PGF_2α_ [89, 90]. Another possible mechanism for preventing luteolysis during maternal recognition of pregnancy is an increase in the PGE_2_:PGF_2α_ ratio in response to estrogen secreted by conceptuses in the uterus [90–94]. Therefore, PG synthesis and secretion appear to be critical and tightly regulated to modulate luteolysis and maternal recognition of pregnancy in the uterine endometrium in pigs.

## Conceptus estrogens and their role in endometrial function

### Conceptus estrogens

It is well established that the elongating conceptus produces estrogens at the time of implantation in pigs, as stated previously [2, 32]. The expression of 17α-hydroxylase (CYP17A1) and aromatase (CYP19A1), enzymes responsible for the synthesis of estrogens, is detectable in the trophectoderm cells of spherical to filamentous conceptuses on d 11 and d 12 [67, 95]. Unconjugated estrogens (estrone, estradiol, and estriol) and sulfur-conjugated estrogens (estrone sulfate, estradiol sulfate and estriol sulfate) are observed in uterine fluids [31, 96]. Estrogen sulfotransferase produced by the endometrium is responsible for the conversion of free estrogens to conjugated estrogens [96, 97]. Catechol estrogens, 2- and 4-hydroxyestradiols, are also produced by the elongating conceptuses, which exhibit estrogen-2-hydroxylase and estrogen-4-hydroxylase activity [34, 35, 98]. In mice, catechol estrogens are involved in the activation of dormant blastocysts for implantation in delayed-implanting mice [99]. Although it has been reported that catechol estrogen induces uterine vasodilation when infused into the utero-artery [100] and changes PG production in cultured endometrial tissues in vitro [101, 102], the role of catechol estrogens in the implantation process is not fully understood in pigs.

### Growth factor expression

The onset of estrogen production by the implanting conceptus coincides with the time of maternal recognition of pregnancy in pigs, and estrogen acts as a maternal pregnancy recognition signal [2, 8]. Conceptus-derived estrogens regulate the expression of a variety of genes involved in cell proliferation, adhesion, migration, PG production, ion and nutrient transport, and immune response in an endometrium primed with progesterone during the implantation period. Many growth factors, including connective tissue growth factor [103], EGF, heparin-binding EGF [64, 104, 105], FGF1, FGF2 [106], and FGF7 [107], IGF1 and IGF2 [108], TGFB1, TGFB2, and TGFB3 [109], and vascular endothelial growth factor [110], are expressed by the endometrium and conceptus during the implantation period and regulate cell division, proliferation, morphogenesis, and differentiation [5]. Among them, the most well-studied growth factors induced by conceptus estrogen during early pregnancy are IGF1 and FGF7. The endometrial transcripts and proteins of IGF1 secreted into the uterine lumen are greatest on d 12 of pregnancy, coincident with maximal estrogen production by the conceptus in pigs [108, 111, 112]. *IGF1* expression is localized in the LE, GE, endothelial, and vascular smooth muscle cells of the endometrium and conceptus trophectoderm [113]; IGF2 is localized in the LE and GE; and IGF-binding protein 2 (IGFBP2) is localized in epithelial and stromal cells [111]. Estrogen injection into ovariectomized pigs and acute estrogen treatment of pigs on d 11 of the estrous cycle increases the endometrial expression and secretion of IGF1 [108]. IGF receptors and IGFBPs regulating the bioavailability of IGFs are expressed by endometrial and conceptus tissues, and IGFBPs are present in the uterine lumen during early pregnancy [67, 111, 114, 115]. It has been shown that IGF1 and IGF2 increase the proliferation of porcine endometrial GE cells in vitro [116]. In addition, it is proposed that IGF1 acts through the stimulation of CYP19A1 expression for conceptus estrogen production based on the overlapping expression patterns of CYP19A1 in the conceptus and IGF concentrations in the uterine lumen [67].

FGF7, also known as keratinocyte growth factor, is a member of the heparin-binding FGF family and stimulates epithelial growth and differentiation [117]. Because FGF7 usually originates from mesenchymal cells and mediates epithelial–mesenchymal interactions in many tissues, including the reproductive tract [117, 118], it was hypothesized that FGF7 is expressed in endometrial stromal cells and regulates epithelial cell function by acting as a progestamedin in the uterine endometrium during the progesterone-dominant period. Contrary to that hypothesis, *FGF7* in the porcine uterus is expressed in endometrial epithelial cells, predominantly in LE cells during early pregnancy and in GE cells during late pregnancy [107]. *FGF7* expression is abundant between d 12 and d 15 of the estrous cycle and pregnancy, with the greatest abundance on d 12 of pregnancy; FGF7 protein is also detectable in uterine flushing on d 12 of both the estrous cycle and pregnancy [107]. Treatment of endometrial explants with estradiol and estradiol injection into ovariectomized pigs increase the expression of *FGF7* in the endometrium, indicating that the dramatic increase in endometrial *FGF7* expression is induced by estrogen of conceptus origin [66, 119]. The FGF7 receptor 2IIIb (*FGFR2IIIb*) is expressed in both the endometrial epithelium and conceptus trophectoderm [107]. Treatment of FGF7 with pTr cells, a trophectoderm cell line derived from d 12 porcine conceptuses, increases [^3^H]thymidine incorporation, phosphorylation of FGFR2IIIb and extracellular signal-regulated kinases 1/2 (ERK1/2), and expression of urokinase-type plasminogen activator (*PLAU*), a marker for differentiation of porcine trophectoderm cells, indicating that FGF7 acts on the proliferation and differentiation of the conceptus trophectoderm in a paracrine manner [66]. The role of FGF7 in endometrial epithelial cells is not yet understood.

### SPP1 expression

The adhesion process between the endometrial epithelium and conceptus trophectoderm requires various cell adhesion molecules to be expressed and produced by the endometrium and trophectoderm [76]. Among the many cell adhesion molecules, SPP1 is the best-characterized molecule to be induced by conceptus-derived estrogen. SPP1, an ECM protein, is a highly phosphorylated acidic glycoprotein that stimulates cell-cell adhesion, increases cell-ECM communication, and promotes cell migration [120]. Endometrial secretion of SPP1 has been shown in several species, including pigs, sheep, humans, nonhuman primates, and rodents [23]. In pigs, *SPP1* expression in the endometrium increases dramatically in LE cells at the time of conceptus implantation. Endometrial LE expression of SPP1 is maintained until late pregnancy, and *SPP1* expression in GE cells is first detected on d 35 and increases thereafter [79]. Estrogen induction of endometrial *SPP1* expression is evidenced by the finding that *SPP1* expression is first detected in endometrial LE cells in direct contact with the implanting conceptus and expands to all LE cells by d 20. Also, injection of estradiol into cyclic pigs to induce pseudopregnancy increases endometrial *SPP1* expression [79, 81]. Immunoreactive SPP1 proteins are found in endometrial LE and GE cells and trophectoderm cells, as well as in uterine flushing [79, 81]. Because SPP1 directly binds to the αvβ6 integrin subunit of pTr cells and the αvβ3 on pUE cells, as noted previously, and because SPP1 can also interact with other integrin receptors, such as α5β1, αvβ1, αvβ5, αvβ6, α8β1, α4β1, α9β1, and α4β7, it is suggested that SPP1 acts as a bidirectional bridging ligand to stimulate cell adhesion, migration, and proliferation for conceptus implantation and placentation [80, 121].

### Calcium secretion and the expression of calcium-regulatory molecules

Calcium plays critical roles in a variety of physiological processes, including bone formation, muscle contraction, and neuronal excitability. At the cellular level, it regulates cell growth, proliferation, differentiation, and death by mediating many cell functions, such as intracellular signaling and cell adhesion [122, 123]. In pigs, it is well established that conceptus estrogen induces endometrial calcium secretion into the uterine lumen during the implantation period; endometrial calcium secretion increases significantly as the conceptuses elongate from tubular to filamentous conceptus stage and decreases by d 14 [29, 88], and endometrial calcium secretion increases in response to estrogen injection into cyclic pigs at 12 h, peaks by 24 h, and declines by 48 h [124, 125]. Although the mechanism underlying estrogen-induced calcium release in the endometrium is not fully understood at the cellular or tissue level in pigs, the expression of calcium extrusion molecules, ATPase Ca^2+^ transporting plasma membrane (also called plasma membrane calcium ATPase), solute carrier family 8 (also called sodium/calcium exchanger), and solute carrier family 24 (also called potassium-dependent sodium/calcium exchanger), in the endometrium indicates that they could be involved in mediating the extrusion of calcium ions across the plasma membranes of cells in the endometrium [126]. During early pregnancy, the expression of stanniocalcin 1 (STC1) has been shown in endometrial LE cells, induced by ovarian progesterone and conceptus estrogen [127], suggesting the possibility of a role for STC1 in endometrial calcium secretion. It is also likely that calcium secretion into the uterine lumen is regulated through a paracellular mechanism at the endometrial epithelial tight junctions, which play a role in the permeability of the paracellular barrier and are differentially expressed in endometrial epithelial cells during early pregnancy in pigs (Choi and Ka, unpublished data).

At the time of implantation in pigs, estrogen also increases endometrial expression of transient receptor potential cation channel subfamily V member 6 (TRPV6), a calcium ion channel responsible for the absorption of calcium ions into the cell, and S100 calcium-binding protein G (S100G, also called calbindin-D9k), an intracellular calcium transport protein [128, 129]. The expression of *TRPV6* and *S100G* has been detected in endometrial LE and trophectoderm cells during early pregnancy, indicating that calcium ions are needed for epithelial and trophectoderm cell functions during the implantation period [128]. Estrogen also increases endometrial calcium absorption in cultured porcine endometrial explant tissues, most likely through TRPV6 (Choi and Ka, unpublished data). The cell adhesion process between endometrial epithelial cells and trophectoderm cells during the implantation period involves many cell adhesion molecules, including integrins, cadherins, selectins, and ECM proteins such as SPP1, which all require calcium ions for appropriate functional activity and are present at the attachment sites at the maternal–conceptus interface in pigs [23, 76]. In addition, it has been shown that the cell adhesion process activates intracellular calcium signaling. Interactions between endometrial epithelial cells and trophoblastic cells in vitro increase calcium influx and intracellular calcium signaling in endometrial epithelial cells in humans [130, 131]. Thus, it is likely that calcium ions secreted by the endometrium and absorbed into endometrial epithelial and conceptus trophectoderm cells play a critical role in the cell adhesion process.

### Regulation of LPA-LPAR3 signaling

**Fig. 2:**
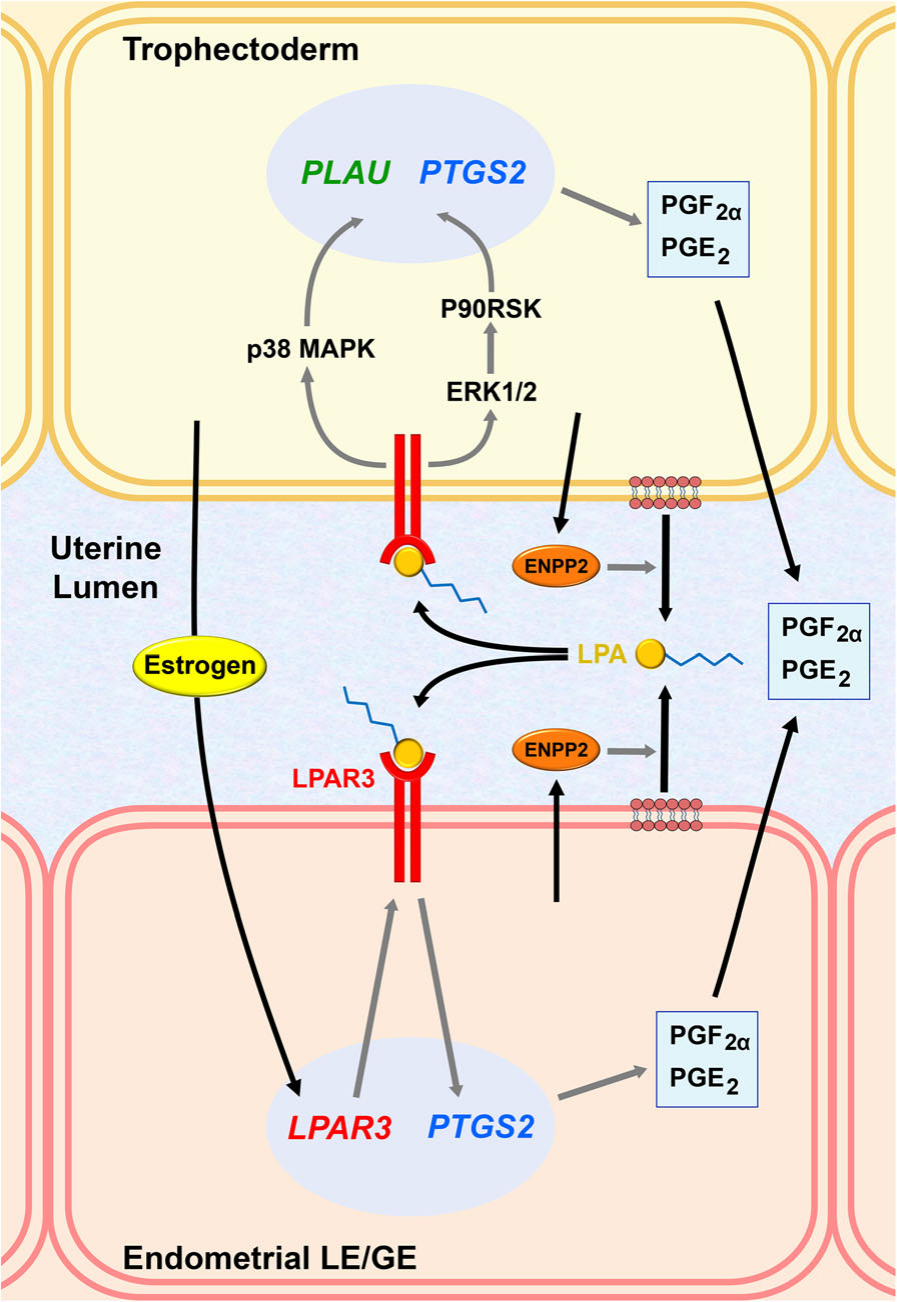
Working model of the role of lysophosphatidic acid (LPA) at the maternal-conceptus interface in pigs. Estrogen of conceptus origin induces endometrial epithelial expression of LPA receptor 3 (LPAR3), and ectonucleotide pyrophosphatase/phosphodiesterase 2 (ENPP2) activates endometrial production of LPA. LPAs secreted into the uterine lumen act on endometrial luminal (LE) and glandular epithelial (GE) cells to increase the expression of prostaglandin (PG)-endoperoxide synthase 2 (PTGS2), which in turn acts on the production of PGF_2α_ and PGE_2_. LPAs also act on the conceptus trophectoderm to activate the extracellular signal-regulated kinases 1/2 (ERK1/2) and p90 ribosomal S6 kinase (P90RSK) signaling pathway and the p38 mitogen-activated protein kinase (MAPK) signaling pathway, which induces the expression of urokinase-type plasminogen activator (PLAU) and PTGS2

Lysophosphatidic acids (LPAs), simple phospholipid-derived mediators, induce many growth factor-like biological effects, such as cell proliferation, survival, migration, and differentiation, via G protein-coupled receptors in various cell types and are found in various body fluids, including serum, saliva, seminal plasma, and follicular fluid [132, 133]. Our study in pigs showed that LPAs (LPA16:0, LPA18:0, LPA18:1, LPA18:2, and LPA20:4) are detectable in uterine lumen, with higher amounts of LPA16:0, LPA18:0, and LPA18:2 on d 12 of pregnancy than on d 12 of the estrous cycle. LPA receptor 3 (LPAR3) is expressed in endometrial epithelial cells, with the greatest abundance on d 12 of pregnancy. In addition, endometrial expression of *LPAR3* is increased by estradiol, indicating that conceptus estrogen is responsible for endometrial *LPAR3* induction [134]. The production of LPAs is mediated by ectonucleotide pyrophosphatase/phosphodiesterase 2 (ENPP2; also called autotaxin), a key enzyme with lysophospholipase D (lysoPLD) activity [135]. In pigs, the uterine endometrium, specifically GE cells, and the conceptus trophectoderm express *ENPP2*, and lysoPLD activity is detected in uterine flushing from d 12 of both the estrous cycle and pregnancy, with higher concentrations on d 12 of pregnancy suggesting the involvement of conceptus signals in increased lysoPLD activity [136]. In mice, deletion of the *Lpar3* gene causes delayed implantation, aberrant embryo spacing, hypertrophic placentas, and embryonic death, along with the reduction of PG-endoperoxide synthase 2 (PTGS2) expression, which results in PGE_2_ and PGI_2_ secretion in the endometrium [137]. In the pig uterus, LPA increases *PTGS2* expression in the endometrium [134]. In a cultured porcine trophectoderm cell line, pTr, LPA activates the ERK1/2 and p90 ribosomal S6 kinase signaling pathway and increases cell proliferation and migration and the expression of *PTGS2* and *PLAU* [138]. The presence of LPA in uterine flushing and LPA-induced increases in cell proliferation and the production of PGE_2_ and PGF_2α_ in trophectoderm cells have been shown in sheep [139]. Overall, these findings indicate that in pigs, conceptus estrogen activates the production of LPA and increased endometrial *LPAR3* expression to regulate endometrial PG production and the proliferation and differentiation of conceptus trophectoderm cells (Fig. 2). Furthermore, because embryo spacing is altered in *Lpar3*-null mice [137], it is likely that the migration and spacing of pig blastocysts, which are critical events preceding implantation and placentation, are also regulated by LPA in pregnant pigs. Recently, it has been reported that *CYP19A1*-null porcine embryos elongate normally but show lowered estrogen production on d 14 postestrus, suggesting that estrogen synthesis is not essential for conceptus elongation [24].

### PG synthesis

PGs derived from the conceptus or endometrium play essential roles in implantation, decidualization, and conceptus development at the maternal-conceptus interface in mammals [140, 141]. In ruminants, IFNT, the maternal pregnancy recognition signal from the conceptus, suppresses the pulsatile release of endometrial PGF_2α_ required for luteolysis by silencing endometrial *ESR1* and *OXTR* expression, although basal concentrations of PGF_2α_ are produced in the endometrium during the implantation period, and PG content in the uterine lumen is much higher during early pregnancy than during the estrous cycle [46, 142]. Conceptus estrogen in pigs increases the production of PGE_2_ and PGF_2α_ in the porcine endometrium [10, 91, 124, 143]. Synthesis of PGs involves the sequential actions of several enzymes, including phospholipase A_2_, PG-endoperoxide synthase 1 (PTGS1), PTGS2, and PG synthases [144, 145]. Aldo-keto reductase 1B1 (AKR1B1) is the major PGF synthase responsible for PGF_2α_ synthesis from PGH2 in bovine and human uterine endometria [146–148]. Our study has also shown that AKR1B1 is responsible for producing PGF_2α_ in the porcine endometrium [10]. Interestingly, *AKR1B1* expression dramatically increases in LE cells of the endometrium on d 12 of pregnancy in pigs, coinciding with conceptus estrogen production [10]. Treatment of endometrial explants with estrogen and estrogen injection into cyclic pigs up-regulate endometrial expression of *AKR1B1* [10, 149], indicating that *AKR1B1* is induced by conceptus estrogen and responsible for increased endometrial production of PGF_2α_.

PG activity during the implantation process includes increased endometrial vascular permeability, endometrial gene expression, and conceptus elongation in many species [94, 140, 141, 150]. In sheep, blocking PG synthesis in the conceptus and endometrium by an intrauterine infusion of meloxicam, a PTGS inhibitor, from d 8 to d 14 post-mating suppresses conceptus elongation on d 14 post-mating, indicating that PGs are essential for conceptus elongation [141]. PGs also regulate the expression of elongation- and implantation-related genes, including *GRP*, *IGFBP1*, *LGALS15*, and *HSD11B1*, in the endometrial epithelium during the implantation period in sheep [142]. In pigs, an intrauterine infusion of PGE_2_ directly inhibits PGF_2α_-induced regression of the CL in a dose-dependent manner, suggesting that PGE_2_ has a luteotrophic effect that protects the CL against the luteolytic action of PGF_2α_ [89, 90, 151]. Recently, Kaczynski and coworkers showed that in pigs, PGF_2α_ induces endometrial expression of vascular endothelial growth factor-A, biglycan, matrix metalloprotease 9, IL1A, and TGFB3, suggesting that PGF_2α_ is involved in angiogenesis and tissue remodeling during early pregnancy [152]. Nevertheless, the detailed functions of PGs at the maternal-conceptus interface in pigs still need further study.

### Regulation of IFN signaling

Conceptus estrogen is also critical to the activation of the endometrial expression of IFN signaling molecules during early pregnancy. Signal transduction and activator of transcription 1 (STAT1) is a key molecule involved in the activation of IFN-stimulated genes (ISGs) in response to type I and II IFNs [153]. *STAT1* expression in the porcine endometrium is detected in LE cells on d 12 of pregnancy and in stromal cells from d 15 of pregnancy [20]. Furthermore, intramuscular estrogen injection into cyclic pigs increases LE expression of *STAT1*, and an intrauterine infusion of conceptus secretory proteins induces stromal expression of *STAT1* [20], indicating that conceptus estrogen and IFNs regulate cell type-specific *STAT1* expression in the endometrium during early pregnancy in pigs. IFN-regulatory factor 2 (*IRF2*), known as a potential transcriptional repressor of ISGs that works by competitively inhibiting IRF1 binding to the promoters of IFN-stimulated responsive elements of ISGs [154], is expressed in endometrial LE cells, with the greatest abundance seen during early pregnancy [19]. Endometrial LE expression of *IRF2* is increased by estrogen, suggesting that IRF2 could suppress the expression of ISGs in endometrial LE cells in pigs [19]. In addition to regulating the expression of intracellular signaling molecules that mediate IFN actions, estrogen also affects the expression of receptors for IFNs in the endometria of pigs. Type I IFNs (including IFND) and type II IFN (IFNG) bind to their heterodimeric type I IFN receptors, IFNAR1 and IFNAR2, and type II IFN receptors, IFNGR1 and IFNGR2, respectively, to transduce signals into the cell [153, 155, 156]. In the porcine endometrium, *IFNAR1* and *IFNAR2* are expressed primarily in LE cells, with the greatest abundance seen on d 12 of pregnancy. The expression of *IFNAR2*, but not *IFNAR1*, is increased by estrogen in endometrial explant cultures [11]. *IFNGR1* and *IFNGR2* are also expressed in the porcine endometrium (endometrial *IFNGR2* expression is greatest on d 12 of pregnancy), and endometrial expression of *IFNGR2*, but not *IFNGR1*, is increased by estrogen in endometrial explant tissues (Choi and Ka, unpublished data). These data suggest that estrogen of conceptus origin induces endometrial expression of IFN receptors to prime the endometrium to respond to IFNs produced by the conceptus during the following few days of estrogen secretion, affecting endometrial function for the establishment of pregnancy.

## Conceptus-derived IL1B and its role in endometrial function

### Conceptus IL1B

IL1B, a well-known pro-inflammatory cytokine, has been shown to play important roles in the implantation process, mediating conceptus-endometrial interactions in several mammalian species, including humans, nonhuman primates, mice, and pigs [3, 157–159]. IL1B production by elongating porcine conceptuses between d 11 and d 12 of pregnancy has been known since the first report of Tuo and coworkers [160]. Recently, Mathew and colleagues have further shown that the *IL1B* gene expressed by porcine conceptuses, *IL1B2*, is different from the classic IL1B gene [4]. The IL1 signaling system consists of two ligands (IL1A and IL1B), two receptors (IL1R1 and IL1R2), an IL1 receptor accessory protein (IL1RAP), and an IL1 receptor antagonist (IL1RN) [161]. IL1R1 is a signaling receptor, whereas IL1R2 is a decoy receptor that does not transduce a signal. A complex composed of IL1B, IL1R1, and IL1RAP is required to initiate IL1B cell signaling. The porcine uterine endometrium expresses *IL1B*, *IL1R1*, *IL1RAP*, and *IL1RN* during the estrous cycle and pregnancy [136, 162]. It has been shown that in pigs, treatment of endometrial tissues with recombinant IL1B2 proteins activates the nuclear factor-kappa B (NFKB) signaling pathway in endometrial epithelial cells [4], and IL1B induces the ERK1/2 and p38 MAPK signaling pathways in the pUE endometrial epithelial cell line [63], indicating that IL1B might activate a wide variety of genes in endometrial epithelial cells during the establishment of pregnancy.

### Regulation of IL1B signaling system

The IL1B receptor subtypes, *IL1R1* and *IL1RAP*, are expressed in the endometrium with the greatest abundance on d 12 of pregnancy, whereas *IL1RN* is expressed at low abundance during early pregnancy in pigs [136, 162]. Endometrial *IL1R1* and *IL1RAP* expression is primarily localized in endometrial LE and GE cells [136]. The great abundance of *IL1B*, *IL1R1*, and *IL1RAP* and low abundance of *IL1RN* at the maternal–conceptus interface during the implantation period suggest that endometrial *IL1R1* and *IL1RAP* expression is regulated by factors of conceptus origin, such as estrogen and IL1B, and that IL1B secreted by the conceptus plays a critical role in implantation by binding to IL1R1 and IL1RAP on the uterine endometrium. Indeed, the results from endometrial explant cultures show that IL1B increases the expression of *IL1R1* and *IL1RAP* in the endometrium of pigs. In addition, estradiol increases the expression of *IL1RAP* in endometrial tissue, indicating that IL1B and estrogen cooperate in the activation of the endometrial IL1B signaling system by activating endometrial *IL1RAP* expression during early pregnancy in pigs [136].

### PG synthesis

The involvement of IL1B in PG production in the endometrium has been shown in several species, including primates, pigs, and ruminants [4, 10, 141, 163–166]. In baboons, IL1B induces the expression of endometrial PTGS2 and IGFBP1 in decidualizing stromal cells to mediate trophoblast invasion and decidualization [163, 164]. In the porcine endometrium, the expression of PG synthetic enzymes is also induced by IL1B [4, 10, 166]. Treating porcine endometrial explant tissue with IL1B or IL1B2 increases the expression of *PTGS1*, *PTGS2*, and *AKR1B1* [4, 10] and the production of PGE [166], suggesting that in addition to conceptus estrogen, IL1B is responsible for the increased endometrial production of PGs in pigs. Recently, it has been indicated that *IL1B2*-null porcine embryos develop normally to the blastocyst stage and form a normal spherical shape but fail to rapidly elongate or survive in utero, with reduced production of estrogen and PGs at the maternal-conceptus interface [24]. IL1B increases the expression of IL1B receptors (*IL1R1* and *IL1RAP*) and *CYP19A1* [4, 10, 166, 167], which indicates that the actions of IL1B are critical for the conceptus-derived production of PGs and estrogen in pigs. In sheep, blocking PG synthesis in the conceptus and endometrium results in the inhibition of conceptus elongation from the ovoidal or tubular form to the filamentous form during early pregnancy, which indicates that PGs are essential for conceptus elongation [141]. However, it is likely that there is no direct effect of PGs on conceptus elongation in pigs, because inhibition of PG synthesis between d 11 to d 12 of pregnancy does not block rapid elongation of conceptuses from spherical to filamentous forms [168].

### PG transport

PGs can cross the cell membrane by simple diffusion at very low amounts but require a facilitated transporter for efficient influx and efflux [169]. The best-characterized PG transporters are the ATP-binding cassette sub-family C member 4 (ABCC4; also known as multidrug resistance-associated protein 4) [170, 171] and solute carrier organic anion transporter family member 2A1 (SLCO2A1; also known as PG transporter). ABCC4 is a transmembrane efflux transporter that can pump its substrates across membranes against a diffusion gradient [171], and SLCO2A1 is responsible for PG influx rather than efflux [172]. The expression of ABCC4 and SLCO2A1 in the endometrium has been shown in several species. ABCC4 is expressed in the bovine endometrium during the estrous cycle and mediates PGF_2α_ and PGE_2_ secretion from endometrial cells [173], and SLCO2A1 is expressed in the uterine endometrium in humans, ruminants, and mice [174–177]. In pigs, endometrial *ABCC4* and *SLCO2A1* expression is biphasic during pregnancy, with the greatest abundance on d 12 and d 90 of pregnancy. IL1B treatment of endometrial explants from d 12 of the estrous cycle increases *ABCC4* and *SLCO2A1* expression [13]. In addition, other possible PG transporters, *ABCC1*, *ABCC9*, *SLCO4C1*, and *SLCO5A1*, are expressed in the porcine endometrium during pregnancy, with the highest expression of *SLCO5A1* on d 12 of pregnancy. The expression of *SLCO4C1* and *SLCO5A1* is increased by IL1B in endometrial tissues in pigs [178]. These data indicate that IL1B derived from the conceptus is involved not only in PG synthesis but also in PG transport in the endometrium during the implantation period in pigs.

ABCC4 and SLCO2A1 are localized at either the apical or basolateral membrane, depending on the cell type [174, 179–181]. Apical localization of ABCC4 in the renal proximal tubule epithelium results in urate exit from the cell into the lumen [181], and SLCO2A1 expressed in the apical membrane of polarized kidney cells is responsible for apical uptake of PGE_2_ [182]. Subcellular localization of those transport proteins seems to be important because it could determine the direction of PG transport. In the porcine endometrium, the expression of ABCC4 is localized mainly in endometrial LE and GE cells, and the expression of SLCO2A1 is localized primarily in endometrial LE and vascular endothelial cells [13]. The pattern of expression and cellular localization of ABCC4 and SLCO2A1 and their mode of action suggest that ABCC4 and SLCO2A1 regulate uterine luminal and utero-ovarian concentrations of PGE_2_ and PGF_2α_, resulting in high concentrations of uterine luminal PGE_2_ and PGF_2α_ and utero-ovarian PGE_2_ at the time of conceptus elongation and the secretion of IL1B and estrogens for pregnancy recognition signaling and implantation. Thus, the location of those PG transporters could be critical for regulating the direction of PG movement in the uterine endometrium as related to the endocrine versus exocrine secretion of PGF_2α_. Nonetheless, the detailed mechanisms of ABCC4 and SLCO2A1 action at the cellular and molecular levels still need further study.

### Salivary lipocalin 1 expression

Lipocalins are a large group of small extracellular proteins that act as transporters of hydrophobic compounds in aqueous biological fluids [183]. The uterine endometrium is known to produce various types of lipocalins, including retinol binding protein in pigs and ruminants [184, 185], uterocalin in mares [186], and lipocalin 2 in mice [187]. Salivary lipocalin (SAL1) is a member of the lipocalin family originally identified as a boar-specific sex pheromone-binding protein [188, 189]; it is also a component of uterine secretions [190]. *SAL1* is expressed in endometrial GE cells at the greatest abundance on d 12 of pregnancy, and endometrial SAL1 protein is secreted into the uterine lumen. *SAL1* expression is increased by IL1B treatment in endometrial explants, indicating that IL1B of conceptus origin induces *SAL1* expression in the endometrium on d 12 of pregnancy [191]. In addition, the abundance of *SAL1* mRNA significantly increases in an endometrium with embryos cloned by somatic cell nuclear transfer compared with an endometrium with normal embryos on d 30 of pregnancy [82]. These data suggest that proper expression of *SAL1* is required for the establishment of pregnancy in pigs. In porcine conceptus tissues on d 12 and d 15 of pregnancy, *SAL1* mRNA is not detectable, but SAL1 proteins are localized in conceptus trophectoderm cells [191], indicating that SAL1 produced in the endometrium using IL1B of conceptus origin transports lipid ligand(s) to the implanting conceptus. Although the identity of the ligand(s) and role of SAL1 at the maternal–fetal interface during the implantation period are not fully understood, the data published so far suggest that SAL1 is a newly identified transport protein that could play a critical role in the establishment of pregnancy in pigs.

### Regulation of IFN signaling molecules

It has been suggested that IL1B plays an important role in the implantation process by regulating the immune response at the maternal–fetal interface [192], but the detailed function of IL1B in the regulation of maternal immune response is not well understood. In humans, IL1 increases production of granulocyte-macrophage colony-stimulating factor in uNK cells, which increase in the endometrium during the mid-secretory phase and contribute a major cellular component of the decidua during pregnancy [193]. Geisert and coworkers have shown that IL1B activates the NFKB signaling pathway in the endometrium [3, 4] and might be involved in activating a variety of cytokines that regulate the maternal immune response in pigs. As previously stated, the porcine endometrium expresses the IFND receptors, *IFNAR1* and *IFNAR2*, in the greatest abundance on d 12 of pregnancy, and IL1B increases the expression of *IFNAR1* and *IFNAR2* in endometrial explant tissues obtained from the uterus on d 12 of the estrous cycle [11], indicating that in addition to estrogen, IL1B is involved in regulating type I IFN receptor expression in the porcine endometrium. IL1B also increases the expression of *STAT1* in endometrial tissues (Choi and Ka, unpublished data). These data suggest that one of the mechanisms by which IL1B regulates the maternal immune response in pigs could be the activation of the IFN signaling pathway.

## Conclusions

**Fig. 3:**
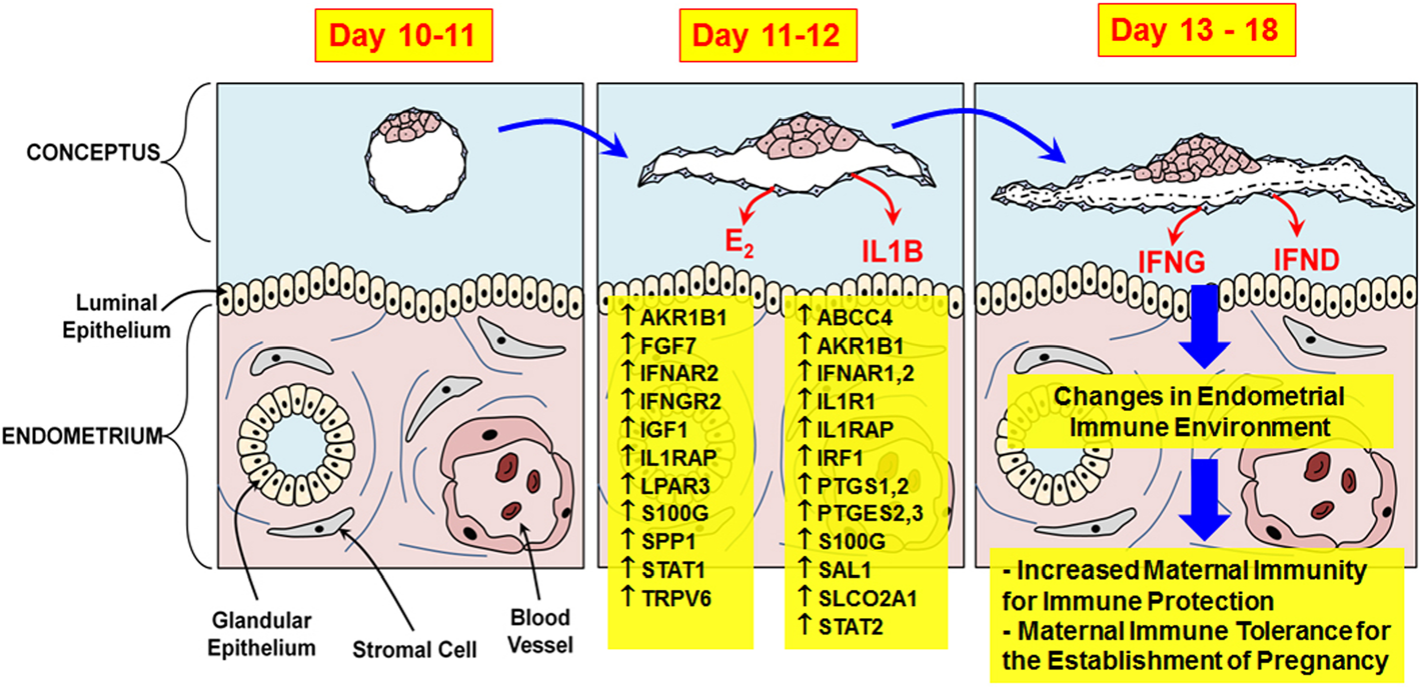
Schematic illustration of the effects of conceptus-derived factors on the expression of genes and possible functions in the endometrium of the porcine uterus during early pregnancy in pigs. Estrogens (E2) and interleukin-1β (IL1B) are secreted by the elongated filamentous conceptus into the uterine lumen on d 11-12 of pregnancy and affect the expression of many endometrial genes, including Aldo-keto reductase 1B1 (AKR1B1), ATP-binding cassette sub-family C member 4 (ABCC4), prostaglandin (PG)-endoperoxide synthases 1 and 2 (PTGS1, PTGS2), and solute carrier organic anion transporter family, member 2A1 (SLCO2A1), that are involved in PG synthesis and transport, leading to the maternal recognition of pregnancy. In addition, E2 and IL1B induce endometrial expression of several interferon (IFN) signaling molecules, including receptors for type I and type II IFNs and IFN-regulatory factor 1 (IRF1) and signal transducers and signal transduction and activator of transcription 1 (STAT1), to prime the endometrium to increase its responsiveness to the actions of IFN-γ (IFNG) and IFN-δ (IFND), which are secreted by the conceptus following its production of estrogen and IL1B on d 12-20 of pregnancy. IFNG and IFND change the endometrial immune environment, increasing maternal immunity for protection and achieving maternal immune tolerance to the semi-allograft conceptus

Establishing a pregnancy requires well-coordinated interactions between the conceptus and the maternal uterine endometrium involving the tightly regulated expression of genes and the production of secretory molecules from the conceptus and the endometrium. Inappropriate interactions result in the failure of normal embryo development and lead to embryonic mortality. This review has focused on the events that occur at the maternal–conceptus interface and the roles of conceptus-derived estrogen and IL1B in endometrial responsiveness during early pregnancy in pigs (Fig. 3). Data from many researchers and our laboratories indicate that estrogen and IL1B derived from elongating porcine conceptuses are involved in cell adhesion and the production of various histotrophs that are essential for the establishment of pregnancy. In particular, estrogen and IL1B cooperate in the endometrial expression of IFN signaling molecules and prime the endometrium to increase its responsiveness to the actions of IFNG and IFND, which are secreted by the conceptus following its production of estrogen and IL1B during early pregnancy. Although we have not discussed the role of conceptus-derived IFNs in this review, those critical immune regulators change the maternal endometrial immune environment to protect the mother and increase tolerance to the semi-allograft conceptus. However, the roles of estrogen and IL1B at the maternal–conceptus interface are far from completely understood and require further analysis. Also, the mechanisms by which IFN activity affects the maternal immune response to achieve immune tolerance to an implanting conceptus for the maintenance of pregnancy need further study in pigs. Studies of the implantation process and the molecules involved provide valuable opportunities to understand the fundamental mechanisms that underlie the establishment of pregnancy in pigs, a species that forms a true epitheliochorial type of placenta.

## Abbreviations

ABCC4: ATP-binding cassette sub-family C member 4;
AKR1B1: Aldo-keto reductase 1B1
CL: Corpus luteum
CYP17A1: 17α-hydroxylase
CYP19A1: Aromatase
ECM: Extracellular matrix
EGF: Epidermal growth factor
ENPP2: Ectonucleotide pyrophosphatase/phosphodiesterase 2
ERK1/2: Extracellular signal–regulated kinases 1/2;
ESR1: Estrogen receptor-α
FGF: Fibroblast growth factor
FGFR: Eibroblast growth factor receptor
FGFR2IIIb: Fibroblast growth factor receptor 2IIIb
GE: Glandular epithelial
IFND: Interferon-δ
IFNG: Interferon-γ
IGF1: Insulin-like growth factor-1
IGFBP2: Insulin-like growth factor -binding protein 2
IL1B: Interleukin-1β
IL1R1: Interleukin-1 receptor 1
IL1RAP: Interleukin-1 receptor accessory protein
IL1RN: Interleukin-1 receptor antagonist
IL6: Interleukin 6
IRF: Interferon-regulatory factor
LAP: Latency-associated peptide
LE: Luminal epithelial
LPA: Lysophosphatidic acid
MAPK: Mitogen-activated protein kinase
MHC: Major histocompatibility complex
MUC1: Mucin 1
NFKB: Nuclear factor-kappa B
PAQR: Progestin and adipoQ receptor
PGE_2_: Prostaglandin E
PGF_2α_: Prostaglandin F_2α_
PGR: Progesterone receptor
PGRMC: Progesterone membrane component
PLAU: Urokinase-type plasminogen activator
PTGS2: Prostaglandin -endoperoxide synthase 2
pTr: Porcine trophectoderm cells
pUE: Porcine uterine endometrial epithelial cells
RGD: Arg-Gly-Asp
S100G: S100 calcium-binding protein G
SAL1: Salivary lipocalin 1
SLCO2A1: Solute carrier organic anion transporter family member 2A1
SPP1: Secreted phosphoprotein 1
STC1: Stanniocalcin 1
TGF: Transforming growth factor beta
TRPV6: Transient receptor potential cation channel subfamily V member 6
